# Random sampling associated with microbial profiling leads to overestimated stochasticity inference in community assembly

**DOI:** 10.3389/fmicb.2022.1011269

**Published:** 2022-10-06

**Authors:** Kai Ma, Qichao Tu

**Affiliations:** ^1^Institute of Marine Science and Technology, Shandong University, Qingdao, China; ^2^Joint Lab for Ocean Research and Education at Dalhousie University, Shandong University and Xiamen University, Qingdao, China

**Keywords:** random sampling, β-diversity, microbial community, stochasticity, null models, Raup–Crick metric, sequencing depth

## Abstract

Revealing the mechanisms governing the complex community assembly over space and time is a central issue in ecology. Null models have been developed to quantitatively disentangle the relative importance of deterministic vs. stochastic processes in structuring the compositional variations of biological communities. Similar approaches have been recently extended to the field of microbial ecology. However, the profiling of highly diverse biological communities (e.g., microbial communities) is severely influenced by random sampling issues, leading to undersampled community profiles and overestimated β-diversity, which may further affect stochasticity inference in community assembly. By implementing simulated datasets, this study demonstrate that microbial stochasticity inference is also affected due to random sampling issues associated with microbial profiling. The effects on microbial stochasticity inference for the whole community and the abundant subcommunities were different using different randomization methods in generating null communities. The stochasticity of rare subcommunities, however, was persistently overestimated irrespective of which randomization method was used. Comparatively, the stochastic ratio approach was more sensitive to random sampling issues, whereas the Raup–Crick metric was more affected by randomization methods. As more studies begin to focus on the mechanisms governing abundant and rare subcommunities, we urge cautions be taken for microbial stochasticity inference based on β-diversity, especially for rare subcommunities. Randomization methods to generate null communities shall also be carefully selected. When necessary, the cutoff used for judging the relative importance of deterministic vs. stochastic processes shall be redefined.

## Introduction

Revealing the mechanisms governing the complex community assembly over space and time is a central issue in ecology. Two distinct types of theories, including the niche theory and the neutral theory (Vandermeer, 1972; Hubbell, 2001), have been developed to explain the compositional variations of biological communities. Historically, the niche theory has gained great success in explaining the dynamic changes in community composition in various ecosystems (Harpole and Tilman, 2007; O'Malley, 2007; Holt, 2009; Kylafis and Loreau, 2011). However, the existence of highly diverse ecosystems such as rainforest, in which many organisms coexist in a same ecological niche (Hubbell, 1979; Scheffer and van Nes, 2006), challenges the throne of niche theory in community ecology. To solve such issues, Hubbell (2001, 2011) proposed the neutral theory, by which many challenges in community ecology can be well resolved. Until now, a general consensus has been reached by ecologists that both deterministic (niche theory) and stochastic (neutral theory) processes shape the assembly of biological communities, but their relative importance may differ in different ecosystems (Dumbrell et al., 2010; Ofiţeru et al., 2010; Chase and Myers, 2011; Stegen et al., 2012; Fisher and Mehta, 2014). Interestingly, recent studies show that sampling scale could be an important factor affecting the relative importance inference of determinism vs. stochasticism in shaping community assembly (Chase, 2014; Correa-Metrio et al., 2014).

Similar issues have been recently recurred in microbial community ecology. Over the last decade, our understanding regarding the complex microbial community assembly has been revisited. For many years, the niche theory has dominated the field with studies mainly focusing on environmental factors that structure the diversity and composition of microbial communities (Fierer and Jackson, 2006; Martiny et al., 2006; Lozupone and Knight, 2007; Fierer et al., 2009; Fuhrman, 2009; Caporaso et al., 2011; Freitas et al., 2012; Thompson et al., 2017; Oliverio et al., 2020). Such efforts can date back as early as to 1930s when Baas Becking proposed the famous hypothesis “Everything is everywhere, but, the environment selects” (Baas-Becking, 1934; Wit and Bouvier, 2006). Important progresses have been made toward our understanding of the relationship between environmental factors and microbial communities. For instance, pH and temperature are found as important factors shaping the diversity and composition of soil microbial communities at large scales (Griffiths et al., 2011; Shen et al., 2013; Sunagawa et al., 2015; Tu et al., 2016; Zhou et al., 2016; Jiao and Lu, 2020; Liang et al., 2020; Xu et al., 2020). Recent studies also demonstrate that both deterministic and stochastic processes play critical roles in structuring the immense microbe world (Zhou et al., 2014; Dini-Andreote et al., 2015; Xue et al., 2018; Nyirabuhoro et al., 2020), and the question to resolve is which process is relatively more important (Antwis et al., 2017; Zhou and Ning, 2017). More recently, studies show that organism size (Farjalla et al., 2012; Wu et al., 2018; Luan et al., 2020) and spatial scale (Shi et al., 2018; Zhang et al., 2020b; Song et al., 2022) are also critical factors influencing the relative importance of deterministic and stochastic processes in structuring microbial communities.

Microbial communities are substantially different from microbial communities regarding the diversity and the role of rare taxa. Typical microbial communities are composed by a small set of abundant taxa and an extremely long tail of rare taxa (Lynch and Neufeld, 2015). The abundant subcommunity usually occupies < 20% of the total richness, but comprises > 80% in relative abundance (Sogin et al., 2006; Lynch and Neufeld, 2015; Zhang et al., 2020a). Notably, studies suggest that the abundant taxa are usually abundant, whereas the rare taxa are persistently rare (Galand et al., 2009). Such scenario also holds true when looking at more systematic microbial community data generated by the Earth Microbiome Project (EMP; Gilbert et al., 2014), the Human Microbiome Project (HMP; Turnbaugh et al., 2007), and the TARA Oceans Expedition (Pesant et al., 2015). Although low in relative abundance, recent studies suggest that the rare subcommunities execute nonnegligible ecosystem functions in the environment (Lyons and Schwartz, 2001; Lyons et al., 2005; Mouillot et al., 2013). For such reasons, efforts have been made to disentangle the underlying ecological mechanisms structuring rare subcommunities (Jia et al., 2018; Mo et al., 2018; Zhang et al., 2018; Jiao and Lu, 2020). Although carried out in different ecosystems, these studies suggest that the abundant and rare subcommunities are structured by different mechanisms (Jia et al., 2018). For instance, the rare subcommunities in subtropical ecosystems are more structured by stochastic processes than abundant subcommunities (Mo et al., 2018; Xue et al., 2018). Similar patterns are also observed for microbial communities in oil-contaminated soils (Jiao et al., 2017). While in the Qinghai-Tibet Plateau wetland ecosystem, it is found that variable selection (deterministic process) governs the community assembly of rare bacteria, whereas dispersal limitation (stochastic process) dominates community assembly of abundant bacteria (Wan et al., 2021).

Notably, the profiling of microbial communities is severely affected by random sampling issues, even using high-throughput sequencing approaches (Zhou et al., 2011, 2013; Zhan et al., 2014b; Tu, 2020). Random sampling issues are associated with each step the microbial communities are profiled, including sample collection, DNA extraction, library construction, amplification, sequencing, and subsequent rarefaction to a same sequencing depth (SeqD). This is mainly caused by the tiny size and high diversity of microbial communities in nature, as well as the limitations of current technologies that complete capturing every single microorganism is not feasible. As a result, only a small portion of the microorganisms in the collected samples are analyzed, leading to undersampled microbial profiles. Specifically, each gram of soil contains as high as 10^4^ prokaryotic species and 10^8^ organisms (Whitman et al., 1998; Torsvik and Øvreås, 2002; Daniel, 2005), while < 100,000 sequences are usually captured for each sample. This number goes much lower after data processing such as quality control and random subsampling/rarefaction to a same SeqD.

In this study, we investigated how microbial stochasticity inference was affected by such undersampled microbial profiles using simulated datasets. Previous studies suggest that random sampling issues associated with microbial profiling lead to overestimated β-diversity (Zhou et al., 2011, 2013; Zhan et al., 2014b; Tu, 2020). And, the effects of random sampling on abundant and rare subcommunities were dramatically different (Zhan et al., 2014a). Because microbial community stochasticity is usually inferred by comparing the observed β-diversity with null expectations, the overestimated β-diversity may lead to more similar/dissimilar patterns with null expectations. Therefore, we expected that microbial stochasticity may also be strongly affected, especially for rare subcommunities. Such effects may differ by different randomization methods generating null communities. By implementing well-controlled simulated datasets, the current study demonstrated solid evidence showing overestimated microbial stochasticity due to random sampling issues associated with microbial profiling, especially for rare subcommunities. Such overestimation eased with increasing SeqD, but could not be eliminated with current sequencing efforts. We therefore urge cautions be made for microbial stochasticity inference using null models.

## Materials and methods

### Methodological framework

**Figure 1:**
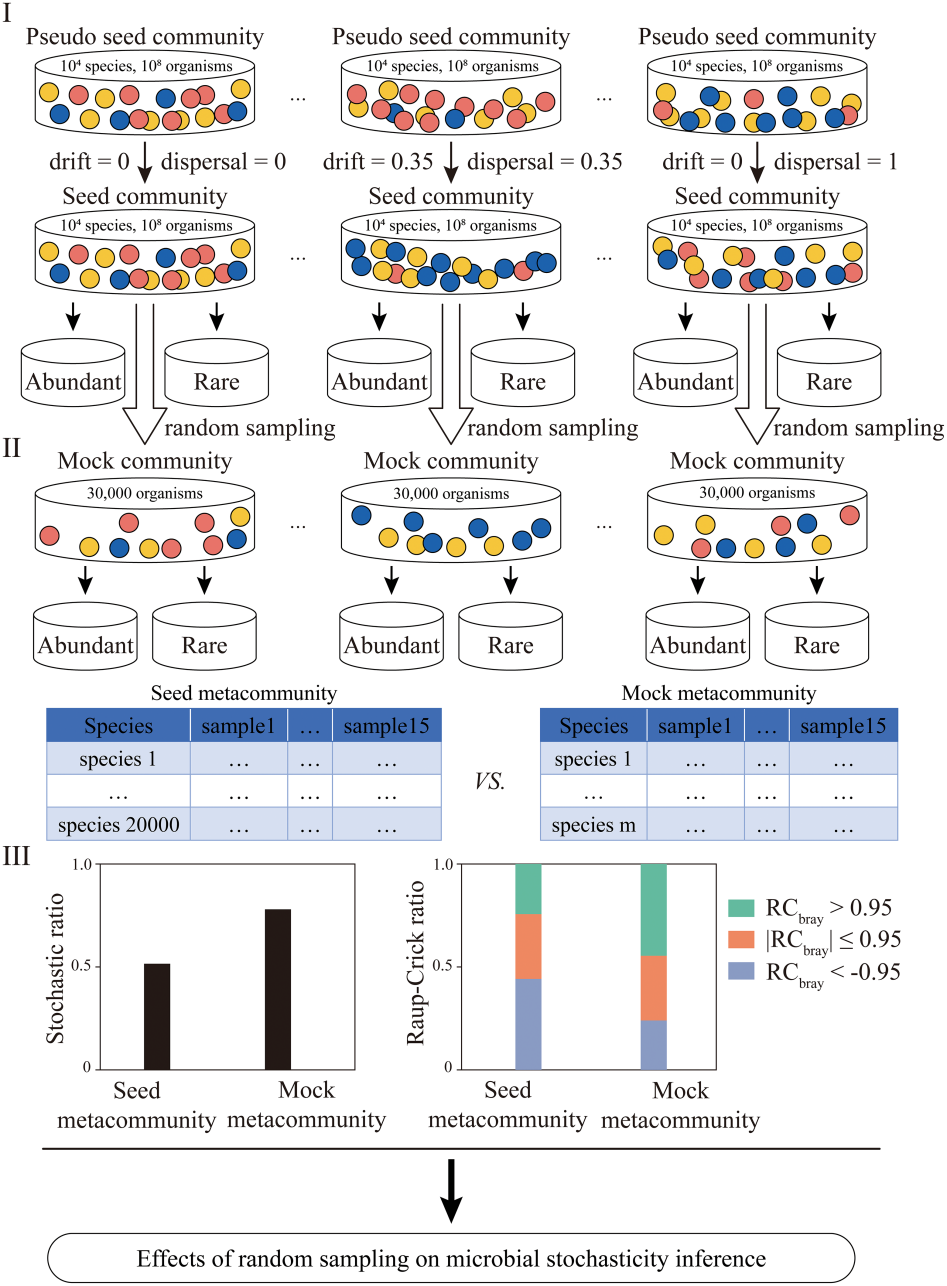
The flowchart for investigating the effects of random sampling issues on microbial stochasticity inference. First, fifteen pseudo seed communities containing 10^4^ microbial taxa and 10^8^ organisms were created. A select portion of microbial taxa were renamed and/or randomly shuffled (Supplementary Table 1), yielding seed communities with different levels of dispersal and drift. Second, mock communities with different sequencing depths were generated by randomly picking 5,000, 10,000, 30,000, 50,000, 70,000, 100,000 and 200,000 sequences from the seed communities. Third, the stochastic ratio and Raup-Crick metric were employed to assess the stochasticity of the seed metacommunity and mock metacommunity, with the difference between them representing the effect of random sampling. Microbial taxa accounting for 80% of the total relative abundance were defined as abundant subcommunity, and the rest were defined as rare subcommunity. The effect of random sampling on abundant and rare subcommunities was also investigated.

A framework was developed to investigate the effects of random sampling issues associated with microbial community profiling on community stochasticity inference (Figure 1). In order to precisely quantify how microbial stochasticity was affected, simulated datasets were constructed and used in this study. First, pseudo seed communities containing 10^4^ microbial taxa and 10^8^ organisms were created. Based on the pseudo seed communities, seed communities with different levels of β-diversity were generated. Then, a seed metacommunity was formed by randomly selecting one of the each seed communities with different diversities and merging them. Second, mock (meta)communities were generated by random subsampling select numbers of organisms from the seed (meta) communities, representing the microbial communities obtained in typical microbial ecological studies. Multiple sets of mock (meta) communities with different organism numbers were generated in order to investigate whether increasing SeqD would eliminate the effects of random sampling issues. Third, microbial profiles were generated for both seed and mock metacommunities, based on which microbial community stochasticity was calculated. The community stochasticity for the seed and the mock metacommunities was comparatively analyzed, with the differences representing the effects of random sampling issues on microbial stochasticity inference. Two different types of stochasticity analyses methods, including the stochastic ratio (Zhou et al., 2014; Zhou and Ning, 2017; Guo et al., 2018) and the Raup–Crick (RC) metric (Raup and Crick, 1979; Chase et al., 2011; Stegen et al., 2013, 2015), were employed here to evaluate how random sampling affected stochasticity inference.

### Seed and mock community construction

A total of 15 pseudo seed communities were constructed following lognormal distributions, which is the species abundance distribution (SAD) model followed by most microbial communities in both natural and artificial ecosystems (Shoemaker et al., 2017). Each seed community was composed by 10^4^ taxa and 10^8^ organisms, representing the basic microbial diversity in per unit environmental samples (e.g., soil; Whitman et al., 1998). A select number (0 to 100%) of taxa in the seed communities were renamed as new taxa and/or randomly shuffled, mimicking community assembly processes such as drift and dispersal limitation. As a result, seed communities with different β-diversity (Bray–Curtis dissimilarity) were generated, and further seed metacommunities were obtained (Supplementary Table 1). Mock (meta)communities were then generated by random subsampling a select number (5 × 10^3^–2 × 10^5^) of organisms from the seed (meta) communities, representing microbial communities under different SeqD. Two major parameters associated with lognormal distribution, including “meanlog” and “sdlog,” were assessed here. The seed metacommunities were found with “meanlog” of 6.80 ± 0.03 and “sdlog” of 2.20 ± 0.00, whereas the values for mock metacommunities (e.g., SeqD = 30,000) were, respectively, 1.00 ± 0.00 and 1.14 ± 0.00 (Supplementary Table 2). These values were comparable to what have been observed for microbial communities in different studies (Supplementary Table 2), such as the Earth Microbiome Project (EMP; Gilbert et al., 2014), the TARA Oceans expedition (Pesant et al., 2015) and the Human Microbiome Project (HMP; Turnbaugh et al., 2007). R packages “mobsim” (May et al., 2018) and a developed R script rarefy_vt.R were, respectively, used for seed community and mock community constructions.

### Defining abundant and rare taxa

No standard is currently available for the definition of abundant and rare microbial taxa in complex communities. Different criteria were used in different studies (Chen et al., 2020; Hou et al., 2020; Jiao and Lu, 2020; Nyirabuhoro et al., 2020). For instance, some studies defined the collection of species with > 0.5% relative abundance as abundant, while the ones with < 0.05% relative abundance as rare (Chen et al., 2020; Hou et al., 2020), whereas in another study the species with > 0.1% relative abundance were considered as abundant and the ones < 0.01% as rare (Jiao and Lu, 2020). In this study, the top ranked microbial taxa who contributed to 80% total relative abundance were defined as abundant, while the rest as rare. Notably, all these criteria satisfy the basic rule of species abundance distribution in community ecology, i.e., the vast majority abundance of microorganisms is dominated by only a few microbial species (Lynch and Neufeld, 2015). Although the abundant and rare taxa identified by different methods may slightly differ, we did not expect strong effect of them on stochasticity analyses.

### Randomization methods to generate null communities

Null models are commonly used to quantitatively disentangle the relative importance of deterministic vs. stochastic processes in structuring the compositional variations of microbial communities. Two different types of randomization methods were employed to generate null communities. The first method shuffles community composition by holding the local diversity and regional diversity close to a constant (Chase et al., 2011; Zhou et al., 2014). Here, the regional species pool is defined as the total number of microbial taxa found in all of the simulated communities with the same SeqD. Dissimilar null communities were expected. The second method draws an individual into a given taxon with a chance proportional to the relative abundance of that taxon in the regional species pool, i.e., all local communities, and at the meanwhile the local diversity and regional diversity are close to a constant (Stegen et al., 2013, 2015). As such, low compositional variations for null communities were expected. The “taxo. Null” function in the R package “NST” was used to generate different types of null communities (Ning et al., 2019). For the first randomization method, parameters including “sp. freq = prop, samp. Rich = fix, abundance = shuffle” were used. For the second randomization method, parameters including “sp. freq = prop, samp. Rich = fix, abundance = region” were used.

### Microbial stochasticity inference using the stochastic ratio approach

Two different approaches were employed to evaluate the effects of random sampling issues on microbial community stochasticity inference. The first one is stochastic ratio analyses (Zhou et al., 2014; Zhou and Ning, 2017; Guo et al., 2018), which was a recently developed approach to quantitatively measure the relative importance of deterministic vs. stochastic processes in structuring the compositional variations of microbial communities. Two kinds of situations were considered in stochastic ratio calculation. First, if communities are governed by deterministic factors leading to more similar communities, the observed community similarity ($C_{ij}$) between the *i*- and *j*-th communities shall be greater than the null expectations ($\bar{E_{ij}}$). Second, if communities are governed by deterministic factors that makes communities more dissimilar, the observed community similarity ($C_{ij}$) between the *i*- and *j*-th communities shall be smaller than the null expectations ($\bar{E_{ij}}$). That being said, the observed dissimilarity ($D_{ij}=1-C_{ij}$) shall be greater than the null model dissimilarity ($\bar{G_{ij}}=1-\bar{E_{ij}}$). The stochastic ratio can therefore be calculated according to the following functions:


$${\mathrm{ST}}_{ij}=\frac{\bar{E_{ij}}}{C_{ij}};\mathrm{if}\;\bar{E_{ij}}<C_{ij}.\left(1\right)\;$$

$$\begin{array}{c} {\mathrm{ST}}_{ij}=\frac{\left(1-\bar{E_{ij}}\right)}{\left(1-C_{ij}\right)};\mathrm{if}\;\bar{E_{ij}}\geq C_{ij}. \end{array}\;\left(2\right)$$

For each type of the abovementioned randomization methods, a total of 1,000 iterations were carried out. The null expectations were calculated by averaging similarity values across these 1,000 null communities. The modified function “tNST” in the R package “NST” to include “shuffle” option in the “abundance” parameter in the source code was used for stochastic ratio analysis (Ning et al., 2019).

### Microbial stochasticity analyses using the RC_bray_ metric

In addition to the stochastic ratio approach, the RC_bray_ metric was also employed to quantify the contribution of different ecological processes to the compositional variations of microbial communities. A similar procedure as described previously was used (Chase and Myers, 2011; Stegen et al., 2013, 2015). Because it was technically almost impossible to simulate the phylogenetic relationships representing the community assembly process of mock communities, null model analysis based on the taxonomic compositional turnover was performed here. Briefly, RC_bray_ values that characterize the magnitude of deviation between the Bray–Curtis dissimilarity values of observed and null communities were calculated. RC_bray_ values larger than 0.95 suggest greater community turnover than null expectations, meaning that deterministic factors that favor different microbes account for the compositional variations. RC_bray_ values smaller than –0.95 suggest less community turnover than null expectations, meaning that deterministic factors that favor similar microbes could be the dominant process for the compositional variations. The fraction of pairwise comparisons with|RC_bray_| ≤ 0.95 suggests comparable community turnover between the observed and null communities, meaning that the compositional variations is a result of stochastic processes. The R function “Raup_Crick_Abundance.r” provided by Stegen et al. (2013)^1^ wasused for RC_bray_ metric analysis.

## Results

### Undersampled microbial profiles dramatically deviated from full profiles.

**Figure 2:**
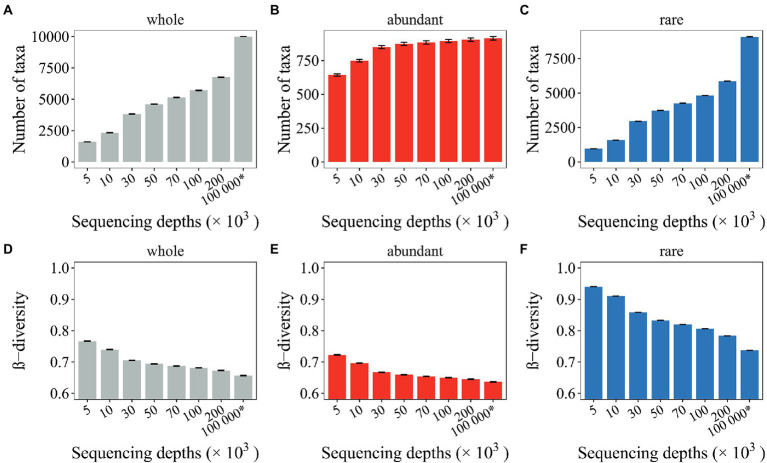
Effects of random sampling issues on the microbial profiles. The number of observed taxa **(A–C)** and the β-diversity **(D– F)** of mock communities with different sequencing depths were investigated. The whole community, the abundant and the rare subcommunities were investigated. The * symbol represents the seed communities consisting of 10^4^ microbial taxa and 10^8^ organisms.

By comparing the compositional variations of mock communities with the seed communities, we investigated whether and how undersampled microbial profiles deviated from full profiles. Here, 15 seed communities following lognormal distribution and with different levels of β-diversity were generated. Each seed community was composed of 10^4^ species and 10^8^ organisms. As a result, seed communities with β-diversity ranging from 0.07 to 0.88 were generated (Supplementary Table 1). Mock communities were then generated by random subsampling a select number of organisms from the seed communities. Here, the seed communities with 0.35 shuffling rate and 0.35 new taxa were randomly selected to illustrate the deviation of undersampled microbial profiles from full profiles (Supplementary Table 1). As a result, a large number of rare taxa (3,228 ~) were not captured by the mock communities, whereas the abundant taxa were rarely affected (Figures 2A–C).

The β-diversity for the seed communities and the mock communities was also comparatively analyzed. Overestimated β-diversity was observed for the undersampled mock communities (~ 0.11), including the whole community, the abundant, and rare subcommunities (Figures 2D–F). Among these, the β-diversity for rare subcommunities (~ 0.20) was the most overestimated (Figure 2F), while the β-diversity for abundant subcommunities (~0.08) was only slightly overestimated (Figure 2E). Notably, increasing SeqD from 50,000 to 200,000 can only slightly ease the situation of overestimated β-diversity (Figures 2D,F), suggesting that the random sampling issues associated with microbial profiling could be persistent with current and near future technologies.

### The β-diversity of null mock communities was also affected

We then investigated how random sampling affected the β-diversity of null communities, based on which microbial stochasticity is inferred. Two types of commonly used randomization methods in microbial community analyses were investigated here.

**Figure 3:**
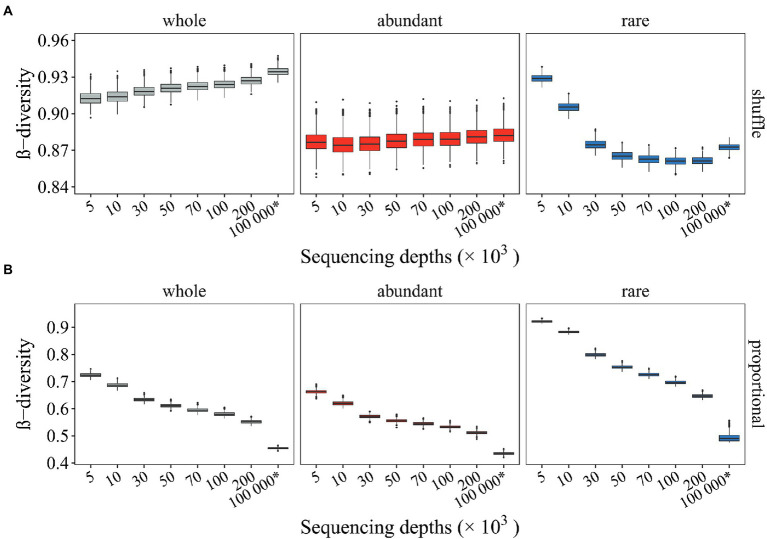
The β-diversity of null communities with different sequencing depth. Null communities were generated by two different types of randomization methods. The β-diversity of the whole community, the abundant and rare subcommunities were investigated. **(A)** The β-diversity of null communities generated by shuffling the community composition by holding the α- and γ-diversity close to a constant (i.e., “shuffle”); **(B)** The β-diversity of null communities generated by drawing an individual into a given taxa proportional to the relative abundance of that taxa in the regional species pool (i.e., “proportional”). The * symbol represents the seed community consisting of 10^4^ microbial taxa and 10^8^ organisms.

As a result, deviated β-diversity of null communities was also observed. Several issues were noticed here (Figure 3). First, as expected, the β-diversity of null communities relative to observed values differed with different randomization methods. For instance, when the community composition was randomly shuffled under constraints, the β-diversity of null communities (Figure 3A) was larger than the observed β-diversity (i.e., whole and SeqD = 5,000: 0.913 > 0.766; Figures 2D–F). However, when the community composition was generated proportionally according to the relative abundance of the taxa in the regional species pool, the β-diversity of null communities (Figure 3B) was much smaller than the observed β-diversity (i.e., whole and SeqD = 5,000: 0.724 < 0.766; Figures 2D–F). Second, the β-diversity of null mock communities relative to that of null seed communities differed with different randomization methods. The β-diversity of null mock communities was smaller than the β-diversity of null seed communities when the community composition was randomly shuffled under constraints (~ 0.927 < 0.935; Figure 3A, “whole”). In contrast, opposite patterns were observed when the randomization of community composition was proportional to the relative abundance of microbial taxa in the regional species pool (0.552 ~ > 0.455; Figure 3B, “whole”). Such different patterns mainly resulted from rare subcommunities, whereas the abundant subcommunities were less affected (Figure 3). Importantly, such thoroughly differed β-diversity of null communities by different randomization methods may result in differed conclusions in microbial community stochasticity inference. Third, samples with low SeqD (e.g., 5,000 and 10,000) deviated more utterly, or even showed opposite pattern (Figure 3). The results suggested that different randomization methods exerted different effects on undersampled microbial profiles, and rare subcommunities were more strongly affected.

### Microbial stochastic ratios were overestimated

**Figure 4:**
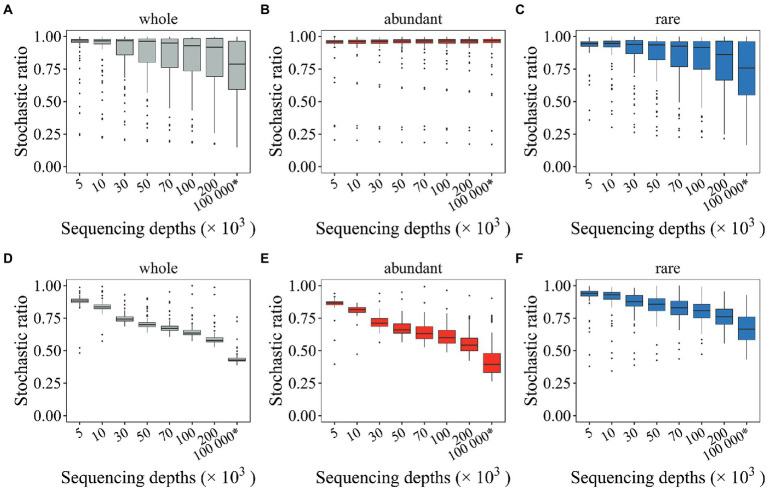
The effect of random sampling on the stochastic ratios of mock metacommunities with different sequencing depths. Two types of randomization methods were investigated, including the “shuffle” **(A–C)** and the “proportional” approach **(D–F)**. The * symbol represents the seed metacommunity consisting of 2 × 10^4^ microbial taxa and 10^8^ organisms.

Multiple community stochasticity inference approaches are available. Here, the stochastic ratio approach (Guo et al., 2018; Ning et al., 2019) was first evaluated to see how undersampled microbial profiles affected microbial community stochasticity. Overestimated stochastic ratio was observed for both randomization methods (Figure 4). Such overestimated stochastic ratio was persistently observed for rare subcommunities regardless of randomization methods (“shuffle” and SeqD = 200,000: 0.796 > 0.724, “proportional” and SeqD = 200,000: 0.765 > 0.671; Figures 4C,F). Comparing to what was observed for rare subcommunities, the effects of random sampling issues on stochastic ratio for abundant subcommunities differ by randomization methods (Figures 4B,E). The stochastic ratio for abundant subcommunities was rarely affected when the “shuffle” randomization method was used (Figure 4B). Most critically, undersampled microbial profiles may lead to dangerously deviated conclusions. For example, when the community composition was randomly shuffled under constraints, high stochastic ratio (> 0.75) was observed for both seed and mock metacommunities (Figures 4A–C). However, when the randomization of community composition was performed by drawing individual organisms proportional to the relative abundance of microbial taxa in the regional species pool, the stochastic ratio was low (~ 0.44) for the seed metacommunity, but high (> 0.59) for mock metacommunities, even for those with 200,000 SeqD (Figure 4D). Such issues also tended to occur with rare subcommunities (Figure 4F). Overall, the results here suggested that undersampled microbial profiles could lead to overestimated stochastic ratio inference, especially for rare subcommunities. Such overestimation may lead to carelessly different conclusions depending on which randomization methods was used.

### Microbial stochasticity inference using the RC_bray_ metric was also affected

**Figure 5:**
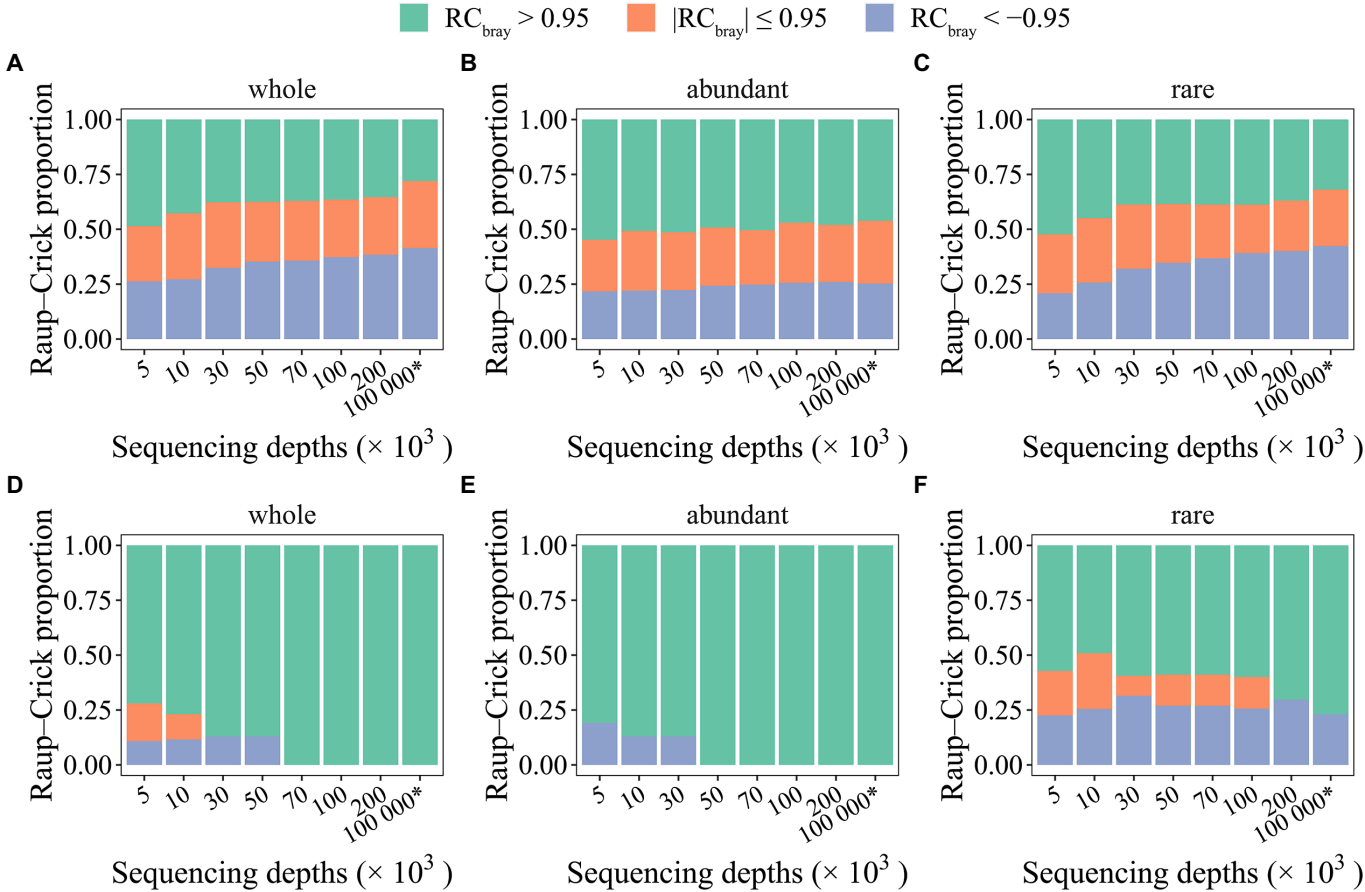
The effect of random sampling on the Raup–Crick metric of mock metacommunities with different sequencing depths. Two randomization methods were used to generate null communities, namely “shuffle” **(A–C)** and “proportional” **(D–F)**. The * symbol represents the seed metacommunity consisting of 2 × 10^4^ microbial taxa and 10^8^ organisms.

In addition to stochastic ratio analyses, the RC_bray_ metric that characterizes the deviation between null distributions and observed taxonomic turnovers to infer the contributions of different processes in community assembly (Stegen et al., 2013, 2015), was also employed to evaluate how stochasticity inference was affected by random sampling issues. Notably, as it was not possible to experimentally generate the required datasets (e.g., deep sequencing of 10^8^ organisms per sample), the same simulated datasets were also used here. And as it was technically almost impossible to simulate the phylogenetic relationships representing the community assembly process of mock communities, the taxonomic compositional turnover was assessed here using the RC_bray_ metric not considering the selection process inferred based on phylogenetic signals. Similarly, the same two different randomization methods (i.e., “shuffle” and “proportional”) were investigated here. Again, thoroughly different results were observed for different randomization methods (Figure 5). Such difference was mainly reflected by the relative contribution of different processes as judged by RC_bray_ values. Notably, when the “shuffle” method was used, the contribution of deterministic factors causing variable communities (RC_bray_ > 0.95) is overestimated, whereas the contribution of deterministic factors causing similar communities (RC_bray_ < −0.95) is underestimated. Such pattern was consistently observed for the whole community, the abundant, and rare subcommunities (Figures 5A–C). However, when the “proportional” randomization method was used, overestimation of stochastic processes was observed for the rare subcommunities (Figure 5F). For the whole and abundant subcommunities, deterministic factors causing variable communities were found as the sole process responsible for the compositional variations of the mock and seed metacommunities when SeqD is larger than 70,000 (Figures 5D,E). The results suggested that RC_bray_ metric is relatively robust to random sampling issues, but could be strongly affected by randomization methods.

## Discussion

Random sampling is a common issue in community ecology as complete sampling is not feasible for large-scale ecosystems or highly diverse communities. This issue becomes more critical in microbial community ecology that almost each step for profiling microbial communities is associated with random processes (Tu, 2020), resulting in undersampled microbial profiles. Previous studies suggest that such random sampling issues affect both the α- and β-diversity estimations of complex microbial communities (Zhou et al., 2011, 2013; Zhan et al., 2014b). The reproducibility could be as low as 17.2% for two technical replicates and 8.2% for three technical replicates, as revealed by 16S rRNA gene amplicon sequencing using 454 pyrosequencing (Zhou et al., 2011). Our recent study suggest that random sampling issues not only affect the α- and β-diversity, but also ecological mechanisms inferred based on these indices, such as spatial scaling laws of microbial communities (Tu, 2020).

In this study, we show that microbial stochasticity inference using null model approaches is also affected by random sampling issues. The inferred community stochasticity for the whole communities, the abundant and the rare subcommunities was all affected due to random sampling issues. This was an especially critical issue for rare subcommunities, whose community stochasticity was persistently thoroughly affected regardless of which null model was used. This was in general consistence with a previous study that random sampling issues mainly affected the reproducibility of rare microbial taxa (Zhan et al., 2014a). As more studies are being made to disentangle the relative importance of deterministic vs. stochastic processes in driving the abundant and rare subcommunities (Jiao et al., 2017; Mo et al., 2018; Xue et al., 2018; Wan et al., 2021), we urge cautions shall be made when interpreting null model results, especially for rare subcommunities.

Different randomization methods to generate null models may lead to different conclusions in microbial stochasticity analyses (Zhou and Ning, 2017). Here, the effects of random sampling issues on microbial community stochasticity inference also thoroughly differ by the randomization methods. Such difference is mainly caused by the fact that microbial stochasticity is inferred by comparing the observed community (dis)similarity with null expectations. The two randomization methods (“shuffle” and “proportional”) used in this study, respectively, generated highly dissimilar and similar null model communities. This consequently led to different conclusions in stochasticity inference. In this study, we found that stochastic ratio approach was more sensitive to random sampling issues than the RC_bray_ approach that overestimated stochastic ratio was observed irrespective of which randomization method was used. In contrast, the RC_bray_ approach was more robust to random sampling issues but more strongly affected by randomization methods. Therefore, proper selection of randomization methods for null models is also strongly recommended.

Mock (meta)communities were generated and used due to the high cost and potential technical barriers of generating the ultra-deep sequence dataset required by this study. The simulated dataset in this study is typical and representative for most environmental samples, although different microbiome types might be differently affected by random sampling issues. For instance, human microbiome that are less diverse than environmental microbiome might less affected (Lozupone et al., 2012). The application of mock (meta)communities allows us to effectively control the variations of microbial communities and generate expected microbial profiles, simulating ecological processes such as drift and dispersal limitation (Ning et al., 2020). However, meanwhile, there are notable caveats associated with simulated datasets. First, as previously pointed out, random sampling is associated with almost all steps microbial profiles are generated, such as sample collection, DNA extraction, PCR amplification, library construction, sequencing and rarefaction (Tu, 2020). Mock (meta) communities, however, are not capable to simulate such complex procedures. In fact, generating mock (meta)communities from seed (meta)communities in the current study could be considered as a unified process anchoring the beginning and ending status of microbial community profiling, leaving the more complex reality not thoroughly considered. Even though, strongly affected microbial stochasticity inference was observed, meaning that the real situation could be much more severe. Secondly, to our best of our knowledge, it was not possible to simulate the phylogenetic relationships representing the complex microbial community assembly processes. Therefore, the current study only considered microbial stochasticity based on taxonomic information, leaving the selection process inferred by phylogenetic signals untapped. Consequently, phylogeny-based β-diversity metrics such as UniFrac (Lozupone and Knight, 2005) and phylo-rpca (Martino et al., 2022) were also not incorporated, though it is relatively easy to incorporate different types of β-diversity metrics in null models. Nonetheless, the obtained results were still informative, showing clearly affected microbial stochasticity inference by random sampling issues associated with microbial community profiling.

Although this study focused on microbial community stochasticity, the ultimate reason causing this scenario was still the overestimated β-diversity by random sampling issues. As a result of random sampling processes associated with microbial profiling, the observed community dissimilarity (i.e., β-diversity) was overestimated, making it closer to the null community compositions. As a result, the stochasticity for the observed communities was overestimated. Because rare subcommunities were more influenced by random sampling issues (Zhan et al., 2014a), the stochasticity of rare subcommunities was more affected than that of abundant subcommunities.

## Conclusion

This study investigated the effects of random sampling issues on microbial stochasticity inference. By implementing simulated datasets, we show evidence that the stochasticity of undersampled microbial communities inferred using null models is overestimated. This issue is especially serious for rare subcommunities. Notably, such effects on the whole community and abundant communities may differ when different randomization methods are used. As more studies begin to focus on the different mechanisms governing the abundant and rare subcommunities, we urge cautions be taken when disentangling the relative importance of deterministic vs. stochastic processes, especially for rare subcommunities. Importantly, such issues could be more severe in reality, as real samples could be far more complex than simulated datasets.

## Data availability statement

All custom scripts and primary data are publicly accessible on GitHub (https://github.com/KaiMa-endeavour/Overestimated-stochasticity).

## Author contributions

QT conceptualized and designed the study. KM analyzed the data and drew the diagrams. QT and KM wrote the manuscript. All authors contributed to the article and approved the submitted version.

## Funding

This study was supported by the National Natural Science Foundation of China (92051110, 31971446), the National Key Research and Development Program of China (2019YFA0606700, 2020YFA0607600), the Natural Science Foundations of Shandong Province (ZR2020YQ21), and the Qilu Young Scholarship of Shandong University. The funders had no role in study design, data collection and interpretation, or the decision to submit the work for publication.

## Conflict of interest

The authors declare that the research was conducted in the absence of any commercial or financial relationships that could be construed as a potential conflict of interest.

## Publisher’s note

All claims expressed in this article are solely those of the authors and do not necessarily represent those of their affiliated organizations, or those of the publisher, the editors and the reviewers. Any product that may be evaluated in this article, or claim that may be made by its manufacturer, is not guaranteed or endorsed by the publisher.

## Footnotes

^1^ https://github.com/stegen/Stegen_etal_ISME_2013
