# Supplementary material for: Random sampling associated with microbial profiling leads to overestimated stochasticity inference in community assembly
Source: Front Microbiol. 2022 Oct 6;13:1011269. doi: 10.3389/fmicb.2022.1011269 (PMC9598869; doi:10.3389/fmicb.2022.1011269)
Supplement: Supplementary file 1 [file Data_Sheet_1.docx]

**Random sampling associated with microbial profiling leads to overestimated stochasticity inference in community assembly**

Kai Ma^1^, Qichao Tu^1,2,*^

^1^ Institute of Marine Science and Technology, Shandong University, Qingdao, 266237, China

^2^ Joint Lab for Ocean Research and Education at Dalhousie University, Shandong University and Xiamen University, Qingdao, China

^*^ Correspondence should be addressed to Qichao Tu ([tuqichao@sdu.edu.cn](mailto:tuqichao@sdu.edu.cn))

**Running title**: Random sampling affects microbial stochasticity

**Supplementary Tables**

[**Supplementary Table 1.** Fifteen seed communities with different levels of β-diversity (Bray-Curtis dissimilarity) were constructed by renaming and/or shuffling a select portion of microbial taxa, mimicking ecological processes such as dispersal and drift. A seed metacommunity was formed by randomly selecting one of each seed community with different diversity and merging them. 3](#_Toc110496612)

[**Supplementary Table 2.** Major parameters of species abundance distribution of simulated communities generated in this study as well as those of real microbial communities in different Earth ecosystems. The lognormal distribution was fitted here. Two major parameters including “meanlog” and “sdlog” were assessed here. EMP: the Earth Microbiome Project; HMP: the Human Microbiome Project. Take a mock metacommunity with a sequencing depth of 30,000 as an example. 4](#_Toc110496613)

**Supplementary Table 1.** Fifteen seed communities with different levels of β-diversity (Bray-Curtis dissimilarity) were constructed by renaming and/or shuffling a select portion of microbial taxa, mimicking ecological processes such as dispersal and drift. A seed metacommunity was formed by randomly selecting one of each seed community with different diversity and merging them.

| **Seed communities** | **Percentage of renamed microbial taxa** | **Percentage of shuffled microbial taxa** | **β-diversity** |
| --- | --- | --- | --- |
| 1st | 0.00 | 0.00 | 0.07±0.00 |
| 2nd | 0.00 | 1.00 | 0.09±0.00 |
| 3rd | 0.05 | 0.05 | 0.21±0.00 |
| 4th | 0.10 | 0.10 | 0.32±0.00 |
| 5th | 0.15 | 0.15 | 0.41±0.00 |
| 6th | 0.20 | 0.20 | 0.49±0.00 |
| 7th | 0.50 | 0.00 | 0.50±0.00 |
| 8th | 0.00 | 0.50 | 0.54±0.00 |
| 9th | 0.25 | 0.25 | 0.56±0.00 |
| 10th | 0.30 | 0.30 | 0.61±0.00 |
| 11th | 0.35 | 0.35 | 0.66±0.00 |
| 12th | 0.40 | 0.40 | 0.69±0.00 |
| 13th | 0.45 | 0.45 | 0.72±0.00 |
| 14th | 0.50 | 0.50 | 0.75±0.00 |
| 15th | 1.00 | 0.00 | 0.88±0.00 |
| seed metacommunity | - | - | 0.90±0.00 |

**Supplementary Table 2.** Major parameters of species abundance distribution of simulated communities generated in this study as well as those of real microbial communities in different Earth ecosystems. The lognormal distribution was fitted here. Two major parameters including “meanlog” and “sdlog” were assessed here. EMP: the Earth Microbiome Project; HMP: the Human Microbiome Project. Take a mock metacommunity with a sequencing depth of 30,000 as an example.

| **data** | **# species (or OTUs)×samples** | **“meanlog”** | **“sdlog”** |
| --- | --- | --- | --- |
| EMP - soil | 3210×3574 | 4.64 | 3.27 |
| EMP - ocean | 1916×1279 | 3.74 | 2.78 |
| TARA Oceans | 4033×139 | 2.23 | 2.34 |
| HMP | 848×4788 | 4.15 | 1.75 |
| seed metacommunity | 20000×15 | 6.80±0.03 | 2.20±0.00 |
| mock metacommunity | 15941×15 | 1.00±0.00 | 1.13±0.00 |
